# Management of hypogammaglobulinemia in pediatric patients with refractory lupus nephritis: a focus on belimumab

**DOI:** 10.3389/fped.2026.1753010

**Published:** 2026-04-22

**Authors:** Yanan Han, Yanjun Yang, Peitong Han, Xiaoying Yuan, Chunzhen Li, Jieyuan Cui

**Affiliations:** Department of Nephrology and Immunology, Children's Hospital of Hebei Province, Hebei Provincial Clinical Research Center for Child Health and Disease, Shijiazhuang, China

**Keywords:** belimumab, hypogammaglobulinemia, level of IgG, lupus nephritis, replacement of IVIG

## Abstract

**Background:**

Although the use of belimumab in children with lupus nephritis (LN) has increased over the past few years, there are limited data on the safety of belimumab in such patients with hypogammaglobulinemia. There are scarce reports of an association between hypogammaglobulinemia and infection in patients with LN who are receiving belimumab treatment.

**Methods:**

We reviewed the cases of 27 patients with lupus nephritis and nephrotic-range proteinuria admitted to Hebei Children's Hospital from January 2019 to October 2022. Among the 27 patients, 12 received intravenous (IV) belimumab (at a dose of 10 mg per kilogram of body weight) plus the standard systemic lupus erythematosus therapy (SoC) (belimumab group) and the other 15 received the SoC (glucocorticoids plus cyclophosphamide or mycophenolate mofetil) (control group). Estimated Systemic Lupus Erythematosus Disease Activity Index (SLEDAI) score; total amount of protein in urine in 24 h; the serum levels of IgG, IgM, IgA, and C3; total B lymphocyte count (BLC); total white lymphocyte count (WBC); erythrocyte sedimentation rate (ESR); and C-reactive protein level were measured five times (at weeks 0, 4, 12, 24, and 52, respectively) in the two groups.

**Results:**

Hypogammaglobulinemia was observed in 22/27 (81.5%) participants with LN prior to initiating treatment. Participants developed hypogammaglobulinemia by week 4. There were no significant differences between the two groups in the total amount of protein in urine in 24 h, serum IgG level, and total B cell count at 0 weeks. Furthermore, no IgG replacement therapy was used in either group until week 15 of observation. However, five patients in the belimumab group and one patient in the control group, whose serum IgG level was below 4 g/L, received 1–2 intravenous immunoglobulin (IVIG) treatments in weeks 16–26 due to severe or recurrent infections. The incidence of infection in the belimumab group was significantly higher than that in the control group, and the IgG serum level in the belimumab group was significantly lower than that in the control group. We also found that the ESR in the belimumab group was significantly lower than that in the control group at weeks 12 and 24 (*P* < 0.05). At weeks 24 and 52, the C3 level in the belimumab group was significantly higher than that in the control group, and the SLEDAI score in the belimumab group was significantly lower than that in the control group (*P* < 0.05). At week 52, the WBC in the belimumab group was significantly higher than that in the control group (*P* < 0.05). However, there was no significant difference in the total amount of protein in urine in 24 h between the two groups at the five time points.

**Conclusions:**

Hypogammaglobulinemia is common in refractory LN. Belimumab treatment may increase the possibility of IgG reduction and the risk of infection. Pediatric patients with LN whose serum IgG levels are below 4 g/L always receive IVIG replacement therapy because of infection. In patients treated with belimumab, monitoring IgG levels is necessary, and IgG replacement therapy should be more aggressive.

## Introduction

Systemic lupus erythematosus (SLE) is a chronic multiorgan autoimmune disease characterized by autoantibody formation, which leads to multisystem inflammation and organ injury (1). Lupus nephritis (LN) is the most common severe manifestation of SLE and a leading cause of illness and death (2). Despite aggressive treatment, the percentage of patients with lupus nephritis who have a positive response remains low, and persistent deterioration eventually progresses to end-stage renal disease. Although immunosuppression regimens, the standard SLE therapy (SoC), including glucocorticoids, cyclophosphamide (CYC), and/or mycophenolate (MMF), have been constantly improving, LN remains a challenge to treat due to a variety of factors (3).

Belimumab (BEL), a humanized monoclonal antibody that binds and neutralizes the B-cell survival factor B lymphocyte stimulator (BLyS), has been extensively studied for the treatment of SLE and LN (4, 5). BLyS (also known as TNFSF13B and BAFF), which induces B-cell proliferation and differentiation, is highly expressed in patients with SLE, thus providing a rationale for developing inhibitors of its function. Given the centrality of BLyS in autoantibody-producing B cells in patients with SLE and LN (6), belimumab is reasonably effective in these patients (7), and belimumab plus the standard therapy is effective in children and adults with SLE (8). However, it is not a panacea for all patients, and the various infections acquired by patients during the belimumab treatment process are one of the main factors affecting the efficacy of LN treatment.

Immunoglobulin abnormalities are common in patients with LN and infection is one of the main factors in the exacerbation of LN. Hypogammaglobulinemia in patients with LN has been attributed to immunosuppressive treatment or effects associated with nephrotic syndrome (9). Patients with LN and hypogammaglobulinemia always receive intravenous immunoglobulin (IVIG) replacement therapy because of recurrent or severe infections (10). However, belimumab therapy may aggravate the risk of infection in patients with LN (11).

There have been no previous reports of an association between belimumab treatment and infection in patients with LN and hypogammaglobulinemia. At present, there are no reports on infections in pediatric patients with hypogammaglobulinemia and lupus nephritis who are receiving belimumab therapy. A phase IV randomized controlled trial in adults conducted by Sheikh et al. reported a serious infection rate of 3.75% in the belimumab group and 4.10% in the placebo group, with no significant difference between the two groups (12). Therefore, we investigated the efficacy and safety of belimumab during treatment of LN, focusing on the relationship between serum IgG levels and serious adverse events such as infections in a real-world setting. We speculate that hypogammaglobulinemia has a predictive role in patients with LN and an infection who are receiving belimumab treatment and that it is an indicator for consideration of IgG replacement therapy.

## Subjects and methods

### Subjects

We reviewed 27 pediatric-onset cases diagnosed with LN and nephrotic-range proteinuria and treated in the Children's Hospital of Hebei Province from January 2019 to October 2025.

The inclusion criteria were as follows: enrolled patients were younger than 18 years of age and had autoantibody-positive SLE (antinuclear antibody titers ≥1:80, anti-double-stranded DNA antibodies, or both) that fulfilled either the 1997 American College of Rheumatology (ACR) classification criteria for SLE, the 2012 Systemic Lupus Erythematosus International Collaborating Clinics (SLICC) criteria, or the 2019 European Alliance of Associations for Rheumatology (EULAR)/ACR classification criteria. The 1997 classification criteria require patients to meet at least four of the 11 criteria. The SLICC criteria require patients to meet at least four of the 17 criteria, including at least one of the 11 clinical standards and one of six immunological criteria, or that the patient has SLE-compliant, biopsy-confirmed nephritis and ANA or anti-dsDNA antibodies. At screening, the patients had a ratio of protein in urine to creatinine of two or more, or a total amount of protein in urine in 24 h (UMTP) of 3.5 g or 50 mg/kg or more. All the patients had biopsy-proven International Society of Nephrology and Renal Pathology Society class III (focal lupus nephritis) or IV (diffuse lupus nephritis) LN with or without coexisting class V (membranous lupus nephritis), or class V lupus nephritis within 6 months before or during screening. Only patients with biopsy specimens showing active lesions or active and chronic lesions were enrolled.

The exclusion criteria included receipt of intravenous immunoglobulin replacement therapy within 3 months before the trial, receipt of B-cell–targeted therapy (including belimumab) within 1 year before, having a congenital immunoglobulin deficiency, receiving dialysis within 1 year, or previous failures of both CYC and MMF.

## Methods

Among the 27 patients, 12 received intravenous (IV) belimumab (at a dose of 10 mg/kg of body weight) plus the SoC (belimumab group) and the other 15 received the SoC (control group). All the patients were free to choose whether to use belimumab. During the trial, the SoC therapy, which may have included glucocorticoids, CYC, and/or MMF, was continued at the discretion of the treating physician.

All clinical data in this study were analyzed and organized using SPSS version 25.0. Continuous variables with a normal distribution are presented as mean ± standard deviation, whereas those not normally distributed are expressed as median and interquartile range. Between-group comparisons (belimumab group vs. control group) were performed using the independent-samples *t*-test; if any variable violated the assumption of normality, the Wilcoxon rank-sum test was applied. A *P*-value < 0.05 was considered statistically significant. Comparisons of indicators at different time points before and after treatment within each group were conducted using one-way repeated-measures analysis of variance, with *P* < 0.05 indicating statistical significance.

In all cases, data acquisition and publication were performed after receiving the patient's or their guardian's written informed consent.

## Results

Baseline characteristics were balanced between the two groups, and there was no difference in the gender, age, or SLEDAI scores of the two cohorts at presentation. The mean (±SD) age was 10.83 ± 2.92 years and 8.87 ± 2.20 years in the belimumab group and control group, respectively, and the SLEDAI score was 19.00 ± 3.69 and 19.40 ± 4.47 at 0 week, respectively (Table 1). There were also no significant differences between the two groups in the total amount of protein in urine in 24 h, serum IgG level, total B-cell count, white blood cell counts, C3 level, and erythrocyte sedimentation rate (ESR) at week 0 (Table 2).

**Table 1:** Baseline demographic and clinical characteristics of the patients.

Characteristic	Belimumab group	Control group	*P*-value
Gender	F/M（5/7）	F/M（6/9）	>0.1
Age (year)	10.83 ± 2.912	8.87 ± 2.20	0.365
SLEDAI score	19.01 ± 3.69	19.40 ± 4.47	0.621

**Table 2:** Data results observed at different weeks.

Time point	Group	UMTP（g/d）	IgG (g/L)	IgM (g/L)	IgA (g/L)	C3 (g/L)	B cell (/µL)	ESR (mm/h)	WBC ( × 10^9^/L)	SLEDAI
0 week	Belimumab	4.78 ± 1.36	3.34 ± 2.00	1.03 ± 0.36	1.62 ± 0.68	0.31 ± 0.12	321.33 ± 84.78	31.75 ± 9.89	8.02 ± 4.58	19.01 ± 3.69
	Control	4.22 ± 1.19	3.20 ± 1.95	1.00 ± 0.33	1.58 ± 0.66	0.31 ± 0.11	326.97 ± 82.89	33.13 ± 11.69	7.80 ± 4.23	19.40 ± 4.47
	P	0.852	0.832	0.877	0.970	0.991	0.885	0.747	0.900	0.621
4 weeks	Belimumab	2.64 ± 0.87	2.95 ± 1.57	1.01 ± 0.307	1.46 ± 0.48	0.48 ± 0.12	331.75 ± 67.48	17.67 ± 7.01	9.22 ± 4.51	10.33 ± 3.89
	Control	2.11 ± 0.80	3.04 ± 1.49	1.01 ± 0.290	1.26 ± 0.49	0.51 ± 0.13	335.27 ± 68.11	17.40 ± 7.70	9.11 ± 4.52	12.47 ± 5.49
	P	0.115	0.873	0.965	0.286	0.596	0.895	0.927	0.953	0.267
12 weeks	Belimumab	1.21 ± 1.12	2.17 ± 0.98	1.02 ± 0.24	1.43 ± 0.61	0.62 ± 0.13	321.70 ± 47.38	7.80 ± 1.40	6.73 ± 2.34	9.01 ± 4.35
	Control	1.13 ± 1.07	3.75 ± 2.02	0.92 ± 0.25	1.28 ± 0.48	0.64 ± 0.12	329.71 ± 63.75	10.29 ± 3.02	6.75 ± 2.22	11.50 ± 5.93
	P	0.986	0.037	0.354	0.521	0.68	0.740	0.025	0.823	0.270
24 weeks	Belimumab	0.44 ± 0.43	6.97 ± 2.53	1.11 ± 0.26	1.46 ± 0.50	1.031 ± 0.20	283.12 ± 27.47	7.25 ± 1.16	6.66 ± 1.29	8.00 ± 4.38
	Control	0.48 ± 0.46	7.89 ± 1.88	1.00 ± 0.20	1.20 ± 0.42	0.76 ± 0.25	309.92 ± 62.77	9.92 ± 2.691	6.32 ± 1.95	10.25 ± 4.88
	P	0.857	0.350	0.318	0.224	0.016	0.271	0.016	0.715	0.010
52 weeks	Belimumab	0.38 ± 0.37	7.25 ± 2.15	1.12 ± 0.13	1.43 ± 0.47	1.15 ± 0.33	270.00 ± 43.91	9.50 ± 3.78	7.00 ± 1.57	6.83 ± 3.82
	Control	0.32 ± 0.30	8.57 ± 1.32	1.04 ± 0.34	1.18 ± 0.37	0.86 ± 0.19	317.17 ± 58.78	11.08 ± 4.40	5.717 ± 0.988	7.50 ± 4.17
	P	0.705	0.126	0.584	0.213	0.029	0.103	0.464	0.048	0.042

Before treatment, 22 of the 27 patients (81.5%) had hypogammaglobulinemia. After the initiation of therapy in these patients with refractory lupus nephritis, the mean serum IgG levels decreased from baseline (week 0) in both groups; however, the differences were not statistically significant (*P* = 0.182 for the belimumab group and *P* = 0.734 for the control group). At week 12, the incidence of infection was significantly higher in the patients in the belimumab group than in the control group, and the IgG serum level was significantly lower in the belimumab group than in the control group (Table 2).

No IVIG treatment was used in either group until the 15th week of observation, but five patients in the belimumab group and one patient in the control group received 1–2 IVIG treatments at week 16–26 due to severe or recurrent infections. Among the five patients in the belimumab group, one patient had severe bacterial pneumonia, one patient had gastrointestinal infection, and three patients had recurrent respiratory infections, while the patient in the control group had recurrent respiratory infections. The IgG level before IVIG treatment was 3.34 ± 2.00 g/L in the belimumab group and 3.20 ± 1.95 g/L in the control group. The serum IgG level in all six patients was below 4 g/L before IVIG treatment. Possibly due to the effect of the IVIG treatment, infection rate and serum IgG level were not significantly different between the two groups at weeks 24 and 52 (Tables 2, 3). No patients received IVIG treatment after week 26.

**Table 3:** Number of infections at different time points.

Time point	Belimumab group	Control group	*P*-value
0W	4/12 (33.3%)	4/15 (26.7%)	0.706
4W	2/12 (16.7%)	2/15 (13.3%）	0.809
12W	4/10 (40%)	1/14（8.3%）	0.049
24W	2/8 (25%)	1/13（7.7%）	0.271
52W	1/6 (16.7%)	1/12（8.3%）	0.596

We also observed that the ESR in the belimumab group was significantly lower than that in the control group at weeks 12 and 24 (*P* < 0.05). At weeks 24 and 52, the C3 level in the belimumab group was significantly higher than that in the control group, while the SLEDAI score in the belimumab group was significantly lower than that in the control group (*P* < 0.05). At week 52, the WBC in the belimumab group was significantly higher than that in the control group (*P* < 0.05). However, there was no significant difference in the total amount of protein in urine in 24 h between the two groups at the five time points (Table 3).

In addition, one-way repeated measures analysis of variance was performed in the belimumab group to evaluate 24-h urinary protein excretion, IgG level, complement C3, erythrocyte sedimentation rate, white blood cell count, and SLEDAI score at weeks 0, 4, 12, 24, and 52. The results showed that 24-h urinary protein excretion and white blood cell count did not meet the assumption of sphericity; therefore, the Greenhouse–Geisser correction was applied. The analyses demonstrated statistically significant differences over time in 24-h urinary protein excretion, IgG level, complement C3, erythrocyte sedimentation rate, and SLEDAI score (*P* < 0.05), whereas no statistically significant difference was observed for white blood cell count (*P* > 0.05). These results are presented in Table 4.

**Table 4:** Observed parameters during treatment in the belimumab group.

Parameter	0W	4W	12W	24W	52W	*F*	*P*-value
UMTP (g/day）	4.78 ± 1.36	2.64 ± 0.87	1.21 ± 1.12	0.44 ± 0.43	0.38 ± 0.37	14.856	0.012
IgG (g/L)	3.34 ± 2.00	2.95 ± 1.57	2.17 ± 0.98	6.97 ± 2.53	7.25 ± 2.15	14.855	0.000
C3 (g/L)	0.31 ± 0.12	0.48 ± 0.12	0.62 ± 0.13	1.031 ± 0.20	1.15 ± 0.33	38.249	0.000
ESR (mm/h)	31.75 ± 9.89	17.67 ± 7.01	7.80 ± 1.40	7.25 ± 1.16	9.50 ± 3.78	12.630	0.000
WBC ( × 10^9^/L)	8.02 ± 4.58	9.22 ± 4.51	6.73 ± 2.34	6.66 ± 1.29	7.00 ± 1.57	1.975	0.190
SLEDAI	19.01 ± 3.69	10.33 ± 3.89	9.01 ± 4.35	8.00 ± 4.38	6.83 ± 3.82	9.389	0.002

## Discussion

Belimumab is a humanized recombinant human IgG-1*λ* monoclonal antibody that inhibits BLyS (also known as B cell-activating factor of the tumor necrosis factor family) and was approved by the US Food and Drug Administration (FDA) and the European Medicines Agency for the treatment of autoantibody-positive SLE with moderate disease activity. Belimumab was recently approved for use in children with SLE between 5 and 17 years of age in China. However, research on Asian populations is scarce.

First, we observed that the serum IgG level initially decreased following treatment in both groups and the patients in both groups developed hypogammaglobulinemia at an early stage. At the initiation of treatment, the infection rates were high in both groups, which may be related to the fact that children with SLE often have immune system dysfunction during the active phase of the disease, leading to reduced host defense against pathogens. In addition, the majority of the patients were in a highly active disease state at enrollment and had already received glucocorticoids and immunosuppressive agents. These medications impair the normal function of neutrophils and lymphocytes, thereby contributing to higher infection rates. At week 12, the incidence of infection was significantly higher in the belimumab group than in the control group, and the IgG serum level in the belimumab group was significantly lower than that in the control group. The incidence of infections in the belimumab group was significantly higher than that in the control group, and the serum IgG level was significantly lower than that in the control group, which may suggest that belimumab therapy is associated with an increased risk of IgG reduction. Inactive lupus nephritis with hypogammaglobulinemia is common and negatively correlated with proteinuria. B cell-related biologics treatment may aggravate the risk of its occurrence. As belimumab is increasingly being used for pediatric LN, including in patients with refractory LN with extensive prior exposure to immunosuppressive treatments (7), the risk of belimumab-related adverse effects may increase, such as hypogammaglobulinemia, which predisposes the patient to recurrent infections, necessitating IVIG supplementation (13). Belimumab binds to BLyS and prevents the binding of soluble BLyS to its receptors on B cells, thereby inhibiting the survival of B cells and reducing the differentiation of B cells into Ig-producing plasma cells (14). This may affect circulating B-cell function and reduce immunoglobulin levels (15). Belimumab treatment appeared to be associated with hypogammaglobulinemia and recurrent infections in our series.

Hypogammaglobulinemia may also be associated with serious infectious adverse events and IVIG replacement can effectively reduce the occurrence of this risk. As a reduced serum IgG level has been reported in refractory LN (16), patients with LN and hypogammaglobulinemia always receive IVIG replacement therapy because of infection and/or risk of infection (11). IVIG therapy has been used to treat a wide variety of immune-driven diseases and severe infections and also as a last-resort therapy to treat organ-specific manifestations of lupus (10). Although studies have shown that belimumab combined with rituximab in the treatment of severe SLE for up to 6.8 years maintained low B-cell counts, no significant hypogammaglobulinemia was observed (17). This does not contradict our results, as the hypogammaglobulinemia we observed in our patients was likely more related to significant proteinuria and not to the use of belimumab. For pediatric patients with refractory LN and extremely low IgG levels and evidence of functional antibody deficiency, especially those treated with belimumab, IVIG replacement treatment can be considered even in the absence of recurrent infection because of the high risk of future infection (18). We found that the serum IgG levels were lowest in the weeks immediately following initiation of induction therapy, and subsequently improved by 24 weeks, and neither infection rates nor serum IgG levels were significantly different between the two groups after this point, which may be related to the administration of IVIG treatment. Thus, the measurement of IgG level is useful in patients with pediatric refractory LN who are receiving belimumab to identify coexisting antibody deficiency and subsequent hypogammaglobulinemia. We suggest assessing serum IgG before commencing treatment, after infusions and before re-treatment, and even in those who develop a recurrent infection. In order to prevent recurrent infections, hypogammaglobulinemia in patients with refractory LN who are receiving belimumab therapy necessitates earlier IgG replacement therapy (19).

In addition, we observed that belimumab treatment was effective in the remission of active pediatric LN after week 24. Moreover, complement C3 was significantly increased and the SLEDAI score was significantly lower in the belimumab group as compared to the control group. However, for pediatric LN with nephrotic syndrome level proteinuria, the treatment effect of total protein in urine was not significantly different between the belimumab group and the control group. This is not completely consistent with some reports. The efficacy of belimumab as an induction treatment in patients with active biopsy-proven LN has been shown in case reports (20). Furie et al. reported that more patients with active LN who received belimumab plus standard therapy had a primary efficacy renal response than those who received standard therapy alone (21). Sciascia et al. reported that the group of patients who received belimumab had a significantly lower risk of annual renal flare rate during the trial than the group of patients who received placebo (22). However, we found that there was no significant difference in proteinuria response between patients with refractory LN who received belimumab and those who did not. These results do not fully align with Furie et al. (21) and may be related to our small sample size.

## Conclusion

In conclusion, belimumab treatment is effective in the remission of active pediatric LN. Hypogammaglobulinemia is common in refractory LN and belimumab treatment may increase the possibility of IgG reduction and the risk of infection. Pediatric patients with LN whose serum IgG level is below 4 g/L always receive IVIG replacement therapy because of infection. In patients treated with belimumab, monitoring IgG levels is necessary, and IgG replacement therapy should be more aggressive.

We recommend the following: (1) obtain a baseline IgG level before starting belimumab treatment in pediatric patients with refractory lupus nephritis, (2) closely monitor for hypogammaglobulinemia after the use of belimumab in pediatric patients, and (3) institute IgG replacement therapy early if patients develop hypogammaglobulinemia (especially when the patient’s serum IgG level is below 4 g/L) in order to prevent recurrent infections.

This study has several limitations. It was a real-world observational study rather than a randomized controlled trial, and therefore potential confounding bias may exist, which could lead to inaccuracies in the assessment of treatment response. In future studies, we plan to expand the sample size, establish case–control groupings, and increase the number of observation time points.

## Data Availability

The original contributions presented in the study are included in the article/supplementary material, further inquiries can be directed to the corresponding author.
